# The Influence of CYP3A4 Genetic Polymorphism and Proton Pump Inhibitors on Osimertinib Metabolism

**DOI:** 10.3389/fphar.2022.794931

**Published:** 2022-03-10

**Authors:** Nanyong Gao, Xiaodan Zhang, Xiaoqin Hu, Qihui Kong, Jianping Cai, Guoxin Hu, Jianchang Qian

**Affiliations:** ^1^ Institute of Molecular Toxicology and Pharmacology, School of Pharmaceutical Sciences, Wenzhou Medical University, Wenzhou, China; ^2^ The Seventh People’s Hospital of Wenzhou, Wenzhou, China

**Keywords:** osimertinib, proton pump inhibitors, CYP3A4, interaction, metabolism

## Abstract

The aim of this study was to 1) investigate the effects of 27 CYP3A4 variants on the metabolism of osimertinib and 2) study the interactions between osimertinib and others as well as the underlying mechanism. A recombinant human CYP3A4 enzymatic incubation system was developed and employed to determine the kinetic profile of CYP3A4 variants. Ultra-performance liquid chromatography–tandem mass spectrometry (UPLC-MS/MS) was applied to detect the concentration of the main metabolite, AZ5104. The results demonstrated that the relative clearance rates of CYP3A4.19, 10, 18, 5, 16, 14, 11, 2, 13, 12, 7, 8, and 17 in catalyzing osimertinib were significantly reduced to a minimum of 25.68% compared to CYP3A4.1, while those of CYP3A4.29, 32, 33, 28, 15, 34, and 3 were obviously enhanced, ranging from 114.14% to 284.52%. The activities of the remaining variants were almost equal to those of CYP3A4.1. In addition, 114 drugs were screened to determine the potential interaction with osimertinib based on the rat liver microsome (RLM) reaction system. Sixteen of them inhibited the production of AZ5104 to 20% or less, especially proton pump inhibitors, among which the IC_50_ of rabeprazole was 6.49 ± 1.17 μM in RLM and 20.39 ± 2.32 μM in human liver microsome (HLM), with both following competitive and non-competitive mixed mechanism. In an *in vivo* study, Sprague–Dawley (SD) rats were randomly divided into groups, with six animals per group, receiving osimertinib with or without rabeprazole, omeprazole, and lansoprazole. We found that the AUC_(0–t)_, AUC_(0–∞)_, and C_max_ of osimertinib decreased significantly after co-administration with rabeprazole orally, but they increased remarkably when osimertinib was administered through intraperitoneal injection. Taken together, our data demonstrate that the genetic polymorphism and proton pump inhibitors remarkably influence the disposition of osimertinib, thereby providing basic data for the precise application of osimertinib.

## Introduction

Osimertinib is an oral, potent, and irreversible epidermal growth factor receptor (EGFR) tyrosine kinase inhibitor, which can selectively and irreversibly inhibit EGFR sensitive mutation of T790M resistant mutation (Planchard et al., 2016; Ricciuti et al., 2017). Currently, it is mainly used in the treatment of patients with non-small cell lung cancer, and it also exerts a good therapeutic effect in patients with resistance to other EGFR tyrosine kinase inhibitors such as gefitinib and afatinib (Jiang and Zhou, 2014; Jänne et al., 2015; Rajappa et al., 2019). It has been shown that osimertinib can significantly prolong the median overall survival of patients (Lee et al., 2021; Tanaka et al., 2021). However, individual differences in its blood concentration, which are mainly caused by genetic polymorphism of metabolic enzyme and drug–drug interactions (DDIs), are one of the most important factors contributing to drug efficacy stratification in the clinical setting.

CYP3A4, a member of cytochrome P450 (CYP450), is the main metabolic pathway involved in the disposition of osimertinib; it generates AZ5104 (desmethyl osimertinib), which accounts for nearly 10% of the prototype drug (Dickinson et al., 2016; Reddy et al., 2018). Any factors that change the metabolic profile of osimertinib would lead to the stratification of drug efficacy. The enzyme activity of CYP3A4 has obvious ethnic variations and is easily influenced by many factors, such as gender, age, and disease state; however, it is mainly influenced by genetic polymorphism, which is responsible for great variability of the enzyme activity among individuals, which further leads to sub-therapeutic phenomena or serious adverse reactions (Nicolas et al., 2009; Roco et al., 2012; Werk and Cascorbi, 2014). Therefore, establishing the association between the genotype and metabolic phenotype of osimertinib is helpful for individualized medication; however, to the best of our knowledge, there is still no related literature. Hitherto, there are 53 CYP3A4 variants that have been identified and named by the Human CYP Allele Nomenclature Committee website (http://www.cypalleles.ki.se/cyp3a4.htm). In this study, we aimed to systematically assess the catalytic activities of wild-type CYP3A4.1 and 26 CYP3A4 variants (including six novel variants discovered by Hu et al.) in the metabolism of osimertinib *in vitro*, so as to provide valuable information for further research (Hu et al., 2017).

DDIs are also an important factor that causes the differences in drug blood exposure. Cancer patients usually experience various complications, such as infections, cardiovascular diseases, and other diseases (Calvo et al., 2019; Ying et al., 2020). Therefore, they often receive multiple drugs concurrently, which may lead to DDIs. In this study, we screened a series of drugs to determine the effect of DDIs on the metabolism of osimertinib. Furthermore, we used rat liver microsomes (RLMs), human liver microsomes (HLMs), and Sprague–Dawley (SD) male rats to study the interaction between osimertinib and proton pump inhibitors (PPIs). The results were expected to provide basic data for promoting precise medical applications of osimertinib.

## Materials and Methods

### Chemicals and Reagents

Osimertinib and AZ5104 were purchased from Beijing Sunflower and Technology Development Co., Ltd. (Beijing, China). Omeprazole, lansoprazole, and rabeprazole were purchased from Shanghai Canspec Scientific Instruments Co., Ltd. (Shanghai, China). Sorafenib was purchased from Shanghai Macklin Biochemical Technology Co., Ltd. (Shanghai, China). Pooled RLMs and HLMs were from Corning Life Sciences Co., Ltd. Recombinant human CYP3A4 and cytochrome b5 were prepared by our group as indicated previously (Zhou et al., 2019). Reduced nicotinamide adenine dinucleotide phosphate (NADPH) was purchased from Roche Pharmaceutical Ltd. (Basel, Switzerland). All other chemicals and solvents not mentioned were of analytical grade. The information on 114 drugs is presented in Supplementary Table S1.

### Equipment and Operating Conditions

Ultra-performance liquid chromatography–tandem mass spectrometry (UPLC-MS/MS) equipped with a Waters Acquity UPLC BEH C18 column (2.1 mm × 50 mm, 1.7-μm particle size; Waters Corp., Millipore, Bedford, MA, USA) was used to detect the concentrations of osimertinib and AZ5104. The temperature of the column and autosampler rack was maintained at 40°C and 4°C, respectively.

The mobile phase consisted of 0.1% formic acid (A) and acetonitrile (B) with gradient elution at 0.4 ml/min for 3.0 min. The following stepwise gradient elution program was used: 90% A (0–0.5 min), 90%–10% A (0.5–1.0 min), 10% A (1–2 min), 10%–90% A (2–2.1 min), and 90% A (2.1–3.0 min). Quantitation was achieved by using a Waters XEVO TQD triple quadruple mass spectrometer. Multiple reaction monitoring (MRM) in the positive mode was selected for detecting the analytes. The monitoring transitions were m/z 500.3 → 385.2, m/z 486.4 → 413.3, and m/z 465.2 → 252.2 for osimertinib, AZ5104, and sorafenib, respectively.

### Kinetic Study of Osimertinib Using Human Recombinant CYP3A4

The 200-μl incubation system consisted of 100 mM of Tris-HCl buffer (pH 7.4), 0.5 pmol of CYP3A4.1 or other CYP3A4 variants, 50 μg/ml of cytochrome b5, 1 mM of NADPH, and 1–100 μM of osimertinib. The mixture without NADPH was pre-incubated at 37°C for 5 min; then, 1 mM of NADPH was added to initiate the reaction. After incubation for 40 min, the reaction was immediately terminated by cooling to −80°C. Then, 400 μl of acetonitrile and 20 μl of sorafenib (100 ng/ml), an internal standard, were added to the mixture. After being vortexed for 2 min and centrifuged at 13,000 rpm for 10 min, the supernatant was obtained for UPLC-MS/MS analysis.

### Determination of Drug–Drug Interactions Using Rat Liver Microsome and Human Liver Microsome

The 200-μl incubation system consisted of 100 mM of Tris-HCl buffer (pH 7.4), 0.2 mg/ml of RLM or HLM, 1 mM of NADPH, and 1–100 μM of osimertinib. When determining the inhibitory effects of 114 drugs on the metabolism of osimertinib, 100 μM of each drug was added to the incubation system, and the volume of buffer was adjusted to maintain the volume of 200 μl. The concentration of osimertinib was set at 25 μM, according to the corresponding *K*_*m*_ (Michaelis–Menten constant) value. The following processing steps were the same as those in the abovementioned experiments. The drugs with an inhibitory rate ≥80% were validated by other independent experimental repeats to confirm the results.

### Inhibitory Effect and the Underlying Mechanism of Proton Pump Inhibitors on Osimertinib in Rat Liver Microsome/Human Liver Microsome

The 200-μl incubation system consisted of osimertinib, PPIs (omeprazole, lansoprazole, and rabeprazole), 100 mM of Tris-HCl buffer (pH 7.4), 0.2 mg/ml of RLM or HLM, and 1 mM of NADPH. In the experiment of half-maximal inhibitory concentration (IC_50_) determination, the concentration of omeprazole or lansoprazole or rabeprazole was set at 0.01, 0.1, 1, 10, 25, 50, and 100 μM, while the concentration of osimertinib was set at 25 μM in RLM and at 40 μM in HLM (according to the corresponding *K*_*m*_ value). To determine the mechanism underlying the inhibitory effect of rabeprazole on osimertinib, the concentration of osimertinib was set at 6.25, 12.5, 25, and 50 μM in RLM and at 10, 20, 40, and 80 μM in HLM according to the *K*_*m*_ value, while the concentration of rabeprazole was set at 0, 3, 6, and 12 μM in RLM and at 0, 10, 20, and 40 μM in HLM according to the IC_50_ value. The following processing steps were the same as those in the abovementioned experiments.

### The Effects of Proton Pump Inhibitors on Osimertinib in Sprague–Dawley Rats

SD male rats (270 ± 10 g) were purchased from the Shanghai Animal Experimental Center. Thirty-six SD rats were divided randomly into six groups (*n* = 6): 4.5 mg/kg of osimertinib by oral administration (Group A); 3.6 mg/kg of omeprazole and 4.5 mg/kg of osimertinib by oral administration (Group B); 2.7 mg/kg of lansoprazole and 4.5 mg/kg of osimertinib by oral administration (Group C); 1.8 mg/kg of rabeprazole and 4.5 mg/kg of osimertinib by oral administration (Group D); 2 mg/kg of osimertinib by intraperitoneal injection (Group E); and 1.8 mg/kg of rabeprazole by oral administration and 2 mg/kg of osimertinib by intraperitoneal injection (Group F). Before the experiments, the rats were fasted for 12 h, with free access to water. When the experiment started, omeprazole, lansoprazole, and rabeprazole, which were dissolved in oil, were orally administered in corresponding groups, while the same dose of oil was administered to Groups A and E. After 30 min, osimertinib was administered to all groups. Blood samples from the tail vein were collected at 0.5, 1, 2, 3, 4, 5, 6, 7, 8, 10, 12, and 24 h in Groups A–D and at 0.33, 0.67, 1, 2, 3, 4, 5, 6, 8, 10, 12, and 24 h in Groups E–F after osimertinib administration. Next, 100 μl of plasma was mixed with 200 μl of acetonitrile and 20 μl of sorafenib (100 ng/ml); after being vortexed for 2 min and centrifuged at 13,000 rpm for 10 min, the supernatant was obtained for UPLC-MS/MS analysis.

### Statistical Analysis

The IC_50_ and Lineweaver–Burk plot were obtained using GraphPad Prism 5.0 software. The pharmacokinetic profiles were explored by employing a non-compartmental analysis with Drug and statistics (DAS) software (Version 3.0, Bontz Inc., Beijing, China). The mean plasma concentration–time curve was generated using Origin 8.0. All data were presented as the mean ± SD and analyzed using SPSS 24.0. One-way ANOVA Dunnett’s test was used to compare parameters of wild-type CYP3A4.1 with those of other variants, and an unpaired *t*-test was applied to compare kinetic parameters among different groups. *p* < 0.05 was considered statistically significant.

## Results

### Development of Ultra-Performance Liquid Chromatography–Tandem Mass Spectrometry to Determine Osimertinib and AZ5104

The liquid chromatogram obtained is shown in Supplementary Figure S1. The retention time of osimertinib, AZ5104, and sorafenib was 1.19, 1.14, and 1.48 min, respectively. All of the substances were effectively separated without mutual interference. The ranges of the standard calibration curves of osimertinib and AZ5104 were both 0.1–500 ng/ml, with correlation coefficients greater than 0.99. The lower limit of quantitation was 0.1 ng/ml for both osimertinib and AZ5104. For validation of the detection method, quality control samples at low, medium, and high concentrations were prepared in six replicates to assess the accuracy, precision, stability, extraction recovery, and matrix effect. The results are shown in Supplementary Tables S2–S4.

### Characterization of the Activities of Recombinant Human CYP3A4 in Disposition of Osimertinib

**FIGURE 1:**
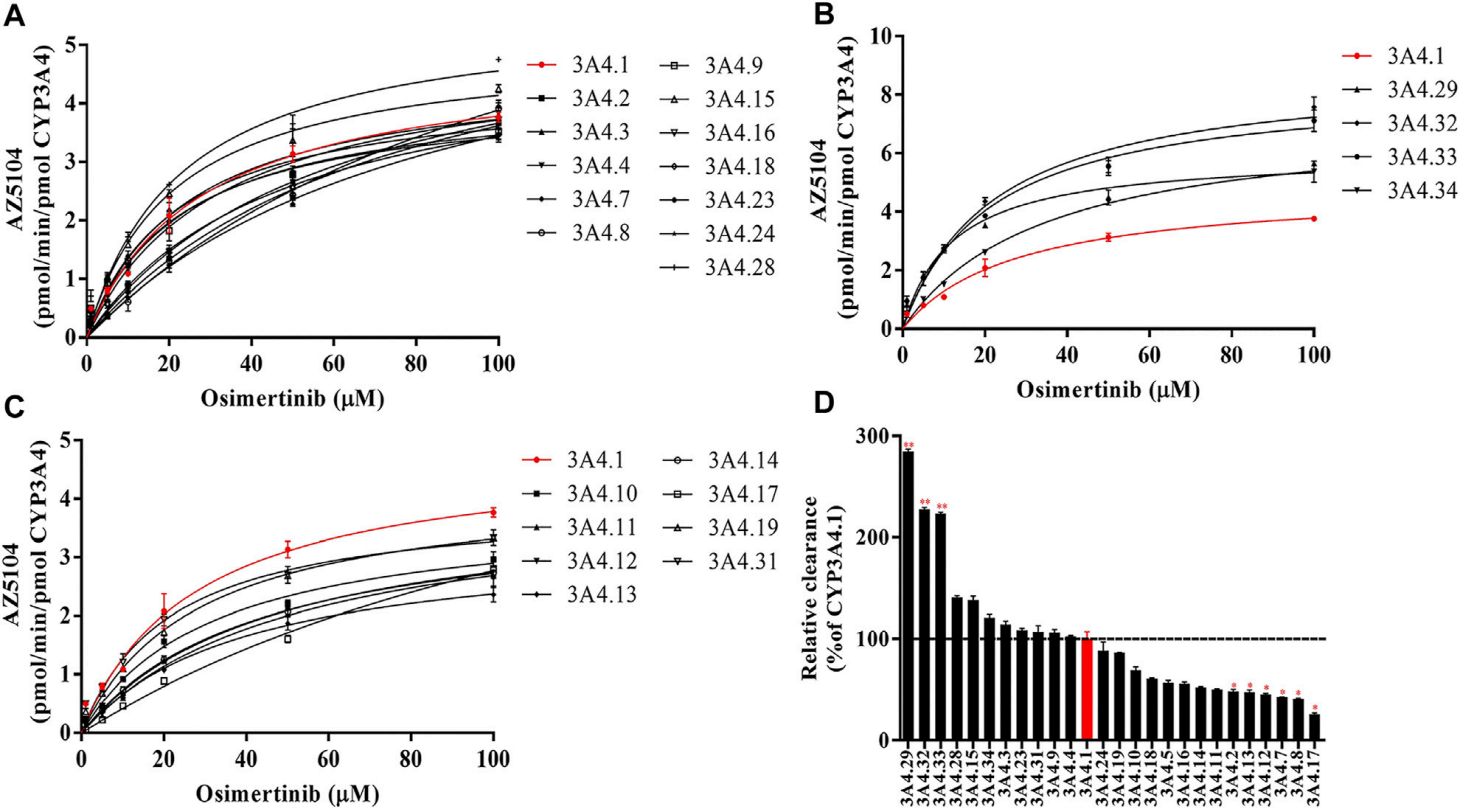
Michaelis–Menten curves of the enzymatic activities of the wild-type CYP3A4 and other CYP3A4 variants in the metabolism of osimertinib **(A–C)** and relative clearance of CYP3A4 variants toward the metabolism of osimertinib compared with the wild type **(D)**. Data are presented as the means ± SD, *n* = 3. *p < 0.05, **p < 0.01, ***p < 0.001.

The Michaelis–Menten curve and the Michaelis kinetic parameters of osimertinib in CYP3A4.1 and other CYP3A4 variants are shown in Figure 1 and Table 1, respectively. According to the alterations in the maximum velocity of the reaction (*V*_*max*_), we observed the following three situations: no obvious differences between CYP3A4.1 and CYP3A4.15, 16, 18, 23, and 24; significant increments in CYP3A4.2, 5, 7, 8, 17, 28, 29, 32, 33, and 34, ranging from 115.66% to 182.70%; and evident decrements in the remaining variants, ranging from 68.98% to 89.75%. According to the alterations in *K*_*m*_, there were also three situations: remarkable increment in CYP3A4.2, 5, 7, 8, 11, 12, 13, 14, 16, 17, and 18, ranging from 145.51% to 584.44% relative to CYP3A4.1; obvious decrements in CYP3A4.15, 29, and 31, ranging from 43.14% to 76.51% as compared with CYP3A4.1; and no significant difference in the remaining variants. The intrinsic clearance (CL_int_) is considered the evaluation criterion for CYP3A4 enzymatic activity. In this study, compared with CYP3A4.1, seven variants showed significant increment (114.14%–284.52%); 12 variants showed obvious decrements (25.68%–69.07%); and the remaining variants showed similar values. Additionally, the concentrations of AZ5104 could not be detected for CYP3A4.20.

**TABLE 1:** Kinetic parameters for AZ5104 activity of CYP3A4.1 and other CYP3A4 variants in osimertinib metabolism.

Variants	*V*_*max*_ (pmol/min/pmol P450)	*K*_*m*_ (μM)	*V*_*max*_/*K*_*m*_ (μl/min/pmol P450)
3A4.1	4.839 ± 0.149	27.890 ± 2.857	0.174 ± 0.012
3A4.2	6.348 ± 0.256*	75.620 ± 6.067*	0.084 ± 0.003*
3A4.3	4.343 ± 0.092	21.830 ± 0.151	0.199 ± 0.006
3A4.4	4.285 ± 0.162	23.970 ± 0.726	0.179 ± 0.002
3A4.5	5.327 ± 0.208	53.797 ± 0.190*	0.099 ± 0.004
3A4.7	6.442 ± 0.076**	86.803 ± 0.829**	0.074 ± 0.000*
3A4.8	8.530 ± 0.144***	119.533 ± 3.553***	0.071 ± 0.001*
3A4.9	4.189 ± 0.154	22.643 ± 0.621	0.185 ± 0.005
3A4.10	3.821 ± 0.221	31.790 ± 2.619	0.120 ± 0.006
3A4.11	4.005 ± 0.023	45.783 ± 0.118	0.087 ± 0.001
3A4.12	4.084 ± 0.282	51.800 ± 2.822*	0.079 ± 0.001
3A4.13	3.338 ± 0.178*	40.583 ± 3.561	0.082 ± 0.004*
3A4.14	3.888 ± 0.031	42.720 ± 0.406	0.091 ± 0.001
3A4.15	4.998 ± 0.129	20.770 ± 0.095	0.241 ± 0.007
3A4.16	5.909 ± 0.818	60.857 ± 8.839	0.097 ± 0.003
3A4.17	7.300 ± 0.437	163.000 ± 2.587***	0.045 ± 0.002*
3A4.18	5.075 ± 0.092	47.750 ± 0.524	0.106 ± 0.001
3A4.19	4.245 ± 0.176	28.183 ± 1.260	0.151 ± 0.001
3A4.20	ND	ND	ND
3A4.23	4.642 ± 0.062	24.557 ± 0.346	0.189 ± 0.003
3A4.24	4.918 ± 0.186	32.283 ± 4.603	0.154 ± 0.015
3A4.28	5.597 ± 0.093	22.807 ± 0.123	0.245 ± 0.003
3A4.29	5.968 ± 0.063*	12.033 ± 0.070*	0.496 ± 0.004**
3A4.31	3.967 ± 0.036	21.340 ± 1.352*	0.186 ± 0.011
3A4.32	8.841 ± 0.212***	22.280 ± 0.398	0.397 ± 0.003**
3A4.33	8.340 ± 0.181***	21.413 ± 0.528	0.389 ± 0.002**
3A4.34	7.266 ± 0.597	34.657 ± 3.845	0.210 ± 0.006

Note. Compared to wild type, *p < 0.05; **p < 0.01; ***p < 0.001.

### Screening of the Drugs That Can Potentially Interact With Osimertinib

**FIGURE 2:**
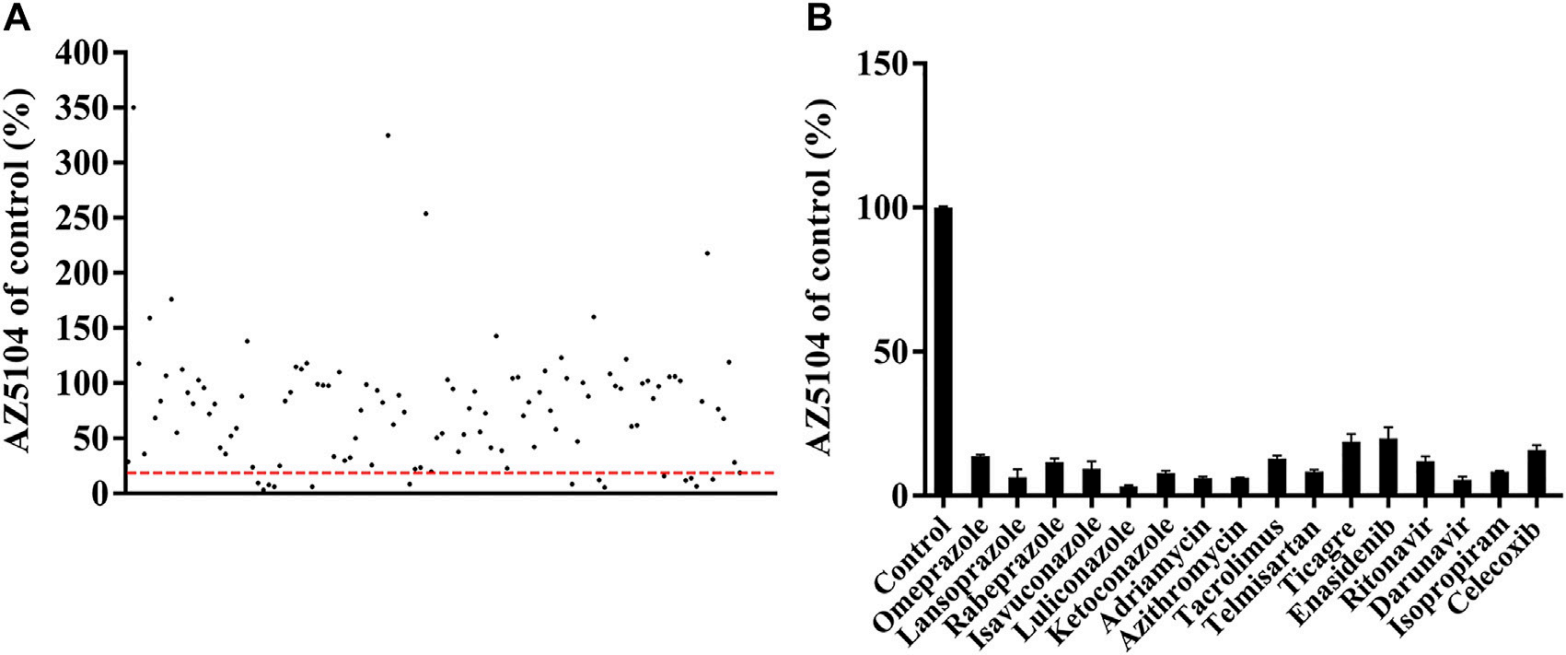
Comparison of the inhibitory effects of drugs (100 μM) on the metabolism of osimertinib in rat liver microsome (RLM). **(A)** All drugs screened. Data are presented as the means. **(B)** The drugs with the inhibition rate >80% are shown. Data are presented as the means ± SD.

The Michaelis–Menten curve for osimertinib in RLM or HLM is shown in Supplementary Figure S2. The values of *V*_*max*_ and *K*_*m*_ in RLM were 0.03 ± 0.00 pmol/min/μg protein and 26.81 ± 2.85 μM, respectively. In HLM, the values of *V*_*max*_ and *K*_*m*_ were 0.05 ± 0.00 pmol/min/μg protein and 41.96 ± 4.39 μM, respectively. The results of an inhibitory effect between osimertinib and other drugs are shown in Figure 2. Among all of the selected drugs, the inhibition rate of the PPIs, namely, omeprazole, lansoprazole, and rabeprazole, on osimertinib reached 86.18%, 93.59%, and 88.33%, respectively, indicating that the combination of PPIs and osimertinib may have a high possibility of a drug interaction.

### Proton Pump Inhibitors Potently Inhibit the Metabolism of Osimertinib in Rat Liver Microsome/Human Liver Microsome With Competitive and Non-Competitive Mixed Mechanism

**FIGURE 3:**
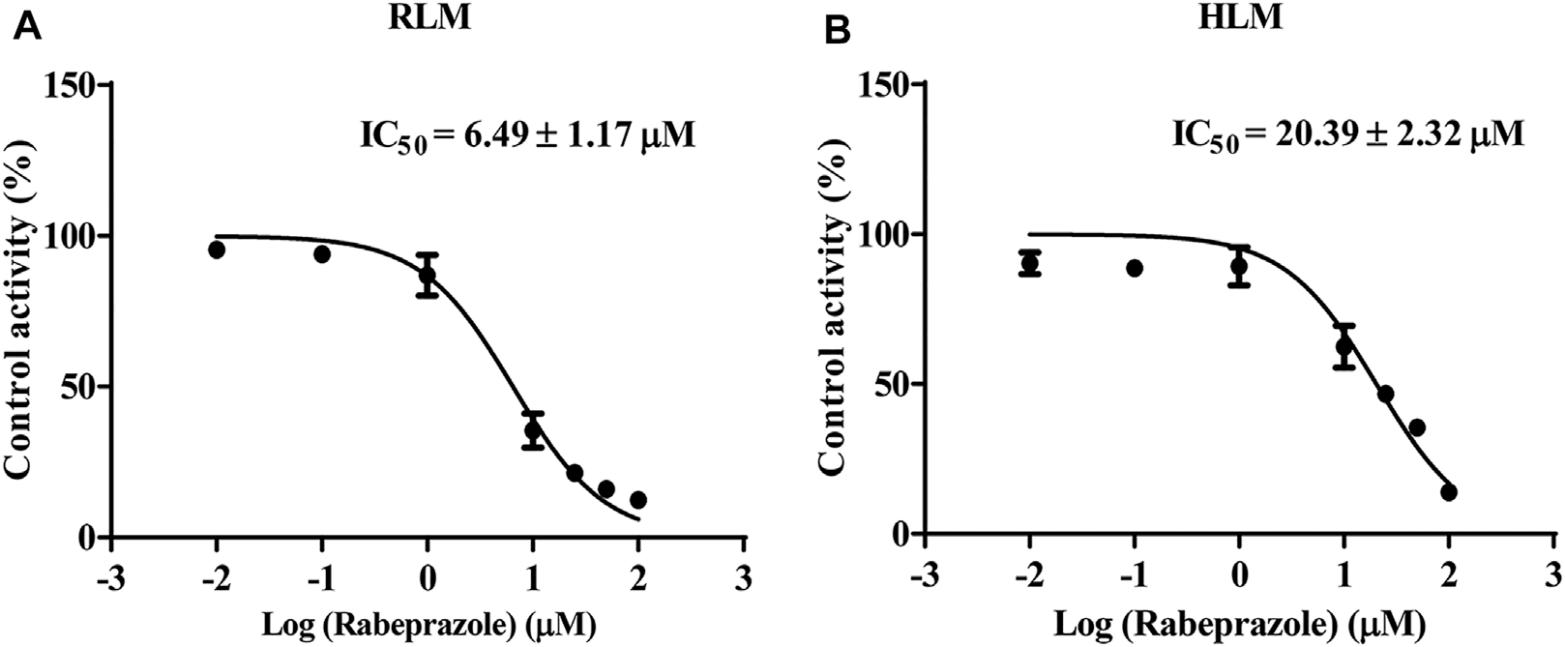
Various concentrations (0.01, 0.1, 1, 10, 25, 50, and 100 μM) of rabeprazole for half-maximal inhibitory concentration (IC_50_) in the activity of **(A)** rat liver microsome (RLM) and **(B)** human liver microsome (HLM). Data are presented as the means ± SD, *n* = 3.

**FIGURE 4:**
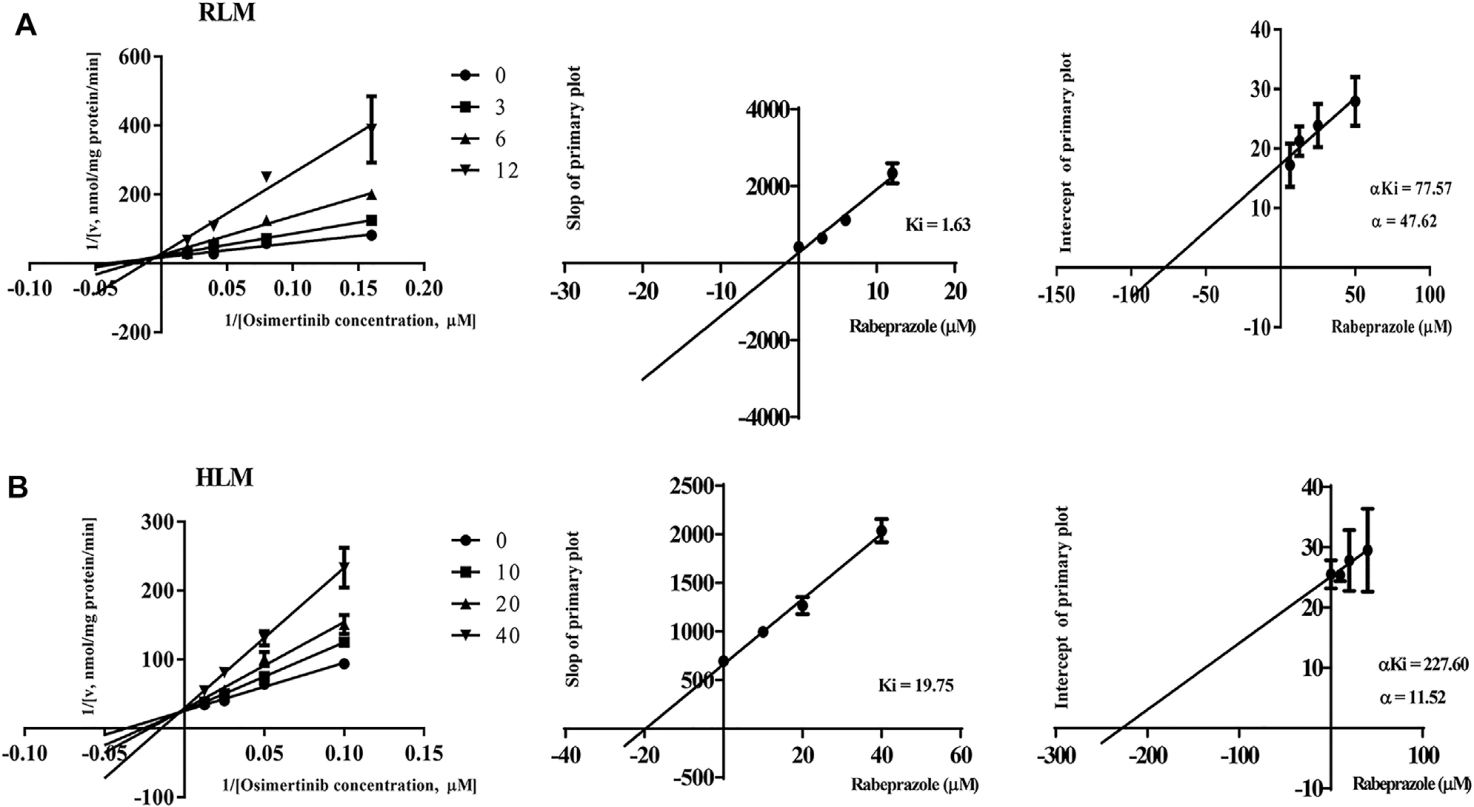
Lineweaver–Burk plot, the secondary plot for *K*_*i*_, and the secondary plot for *αK*_*i*_ for the inhibition of osimertinib metabolism by rabeprazole with various concentrations in **(A)** rat liver microsome (RLM) and **(B)** human liver microsome (HLM). Data are presented as the means ± SD, *n* = 3.

The IC_50_ curves and Lineweaver–Burk plots of rabeprazole on the metabolism of osimertinib are shown in Figures 3, 4. The results indicated that rabeprazole had a strong inhibitory effect on osimertinib, with the IC_50_ values of 6.49 ± 1.17 and 20.39 ± 2.32 μM in RLM and HLM, respectively. The results also showed that rabeprazole can inhibit the metabolism of osimertinib in a mixed way in both RLM and HLM. In addition, we determined the IC_50_ values of omeprazole and lansoprazole on osimertinib in RLM, and the results are shown in Supplementary Figure S3.

### Proton Pump Inhibitors Change the Main Pharmacokinetic Profile of Osimertinib in Sprague–Dawley Rats

**FIGURE 5:**
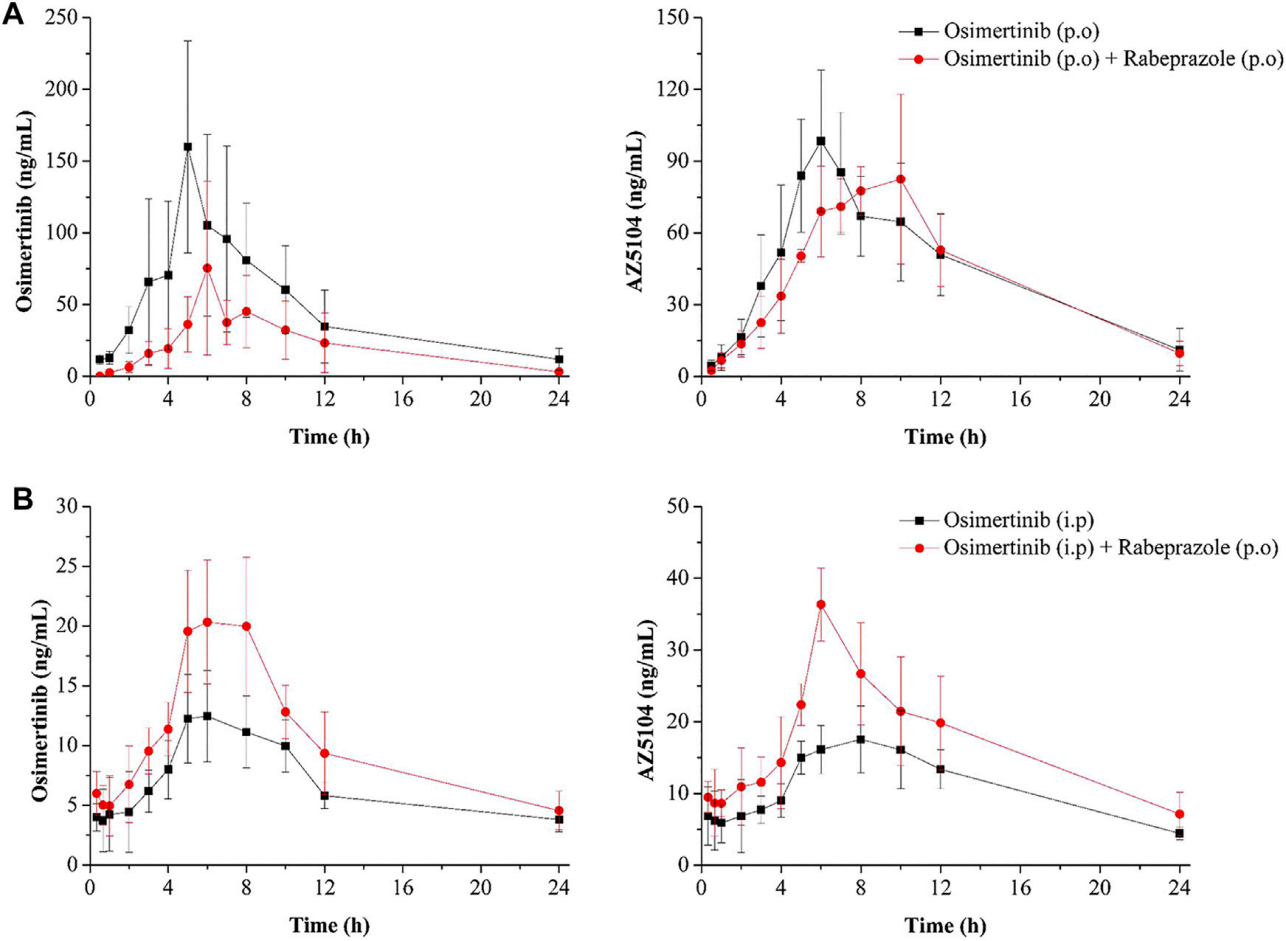
Mean concentration–time curve of osimertinib and AZ5104. **(A)** Osimertinib (p.o.) and osimertinib (p.o.) with rabeprazole (p.o.). **(B)** Osimertinib (i.p.) and osimertinib (i.p.) with rabeprazole (p.o.). Data are presented as the means ± SD, *n* = 6.

The mean concentration–time curves of osimertinib and AZ5104 are shown in Figure 5, and the corresponding pharmacokinetic parameters are shown in Tables 2–5. When rabeprazole and osimertinib were both administered orally, compared with the control group, the values of AUC_(0–t)_, AUC_(0–∞)_, and C_max_ decreased to 42.98%–57.08% for osimertinib, while the parameters of AZ5104 showed no significant difference. When osimertinib was administered by intraperitoneal injection and rabeprazole was orally administered, compared with the control group, the values of AUC_(0–t)_, AUC_(0–∞)_, and C_max_ increased to 125.56%–162.77% for osimertinib and to 145.40%–151.20% for AZ5104. In addition, we also studied the effects of omeprazole and lansoprazole on osimertinib with all drugs administered orally, and the results are shown in Supplementary Figure S4, Supplementary Tables S5, S6. The results were similar to those of rabeprazole, indicating that the three drugs may have similar effects on osimertinib.

**TABLE 2:** The main pharmacokinetic parameters of osimertinib in two groups of rats (*N* = 6).

Parameters	Osimertinib (p.o.)	Osimertinib (p.o.) + rabeprazole (p.o.)
AUC_(0–t)_ (μg/L·h)	1,105.401 ± 548.833	553.775 ± 229.764*
AUC_(0–∞)_ (μg/L·h)	1,208.097 ± 590.583	689.543 ± 195.231*
MRT_(0–t)_ (h)	8.268 ± 1.148	9.203 ± 2.251
MRT_(0–∞)_ (h)	10.085 ± 2.744	12.110 ± 2.499
t_1/2z_ (h)	5.198 ± 2.771	4.533 ± 2.559
T_max_ (h)	5.333 ± 1.033	6.833 ± 0.753
V_z/F_ (L/kg)	30.519 ± 14.042	42.479 ± 21.852
CL_z/F_ (L/h/kg)	4.513 ± 2.040	6.999 ± 2.060
C_max_ (μg/L)	163.042 ± 66.742	70.079 ± 16.770*

Note. AUC, area under the blood concentration–time curve; MRT, mean retention time; *t*_1/2z_, elimination half time; *T*_max_, peak time; *V*_*z*/F_, apparent volume of distribution; *CL*_z/F_, blood clearance; *C*_max_, maximum blood concentration.

*p < 0.05, in comparison with the control group.

**TABLE 3:** The main pharmacokinetic parameters of AZ5104 in two groups of rats (*N* = 6).

Parameters	Osimertinib (p.o.)	Osimertinib (p.o.) + rabeprazole (p.o.)
AUC_(0–t)_ (μg/L·h)	1,044.596 ± 260.978	1,092.676 ± 429.960
AUC_(0–∞)_ (μg/L·h)	1,118.363 ± 301.951	1,109.872 ± 322.543
MRT_(0–t)_ (h)	9.537 ± 0.905	9.967 ± 0.279
MRT_(0–∞)_ (h)	10.912 ± 1.657	13.901 ± 6.240
t_1/2z_ (h)	5.010 ± 1.197	4.740 ± 0.779
T_max_ (h)	6.667 ± 1.633	8.167 ± 1.602
V_z/F_ (L/kg)	30.203 ± 7.879	27.969 ± 15.391
CL_z/F_ (L/h/kg)	4.267 ± 1.093	3.921 ± 1.839
C_max_ (μg/L)	103.368 ± 29.188	94.829 ± 28.781

Note. AUC, area under the blood concentration–time curve; MRT, mean retention time; *t*_1/2z_, elimination half time; *T*_max_, peak time; *V*_z/F_, apparent volume of distribution; *CL*_z/F_, blood clearance; *C*_max_, maximum blood concentration.

**TABLE 4:** The main pharmacokinetic parameters of osimertinib in two groups of rats (*N* = 6).

Parameters	Osimertinib (i.p)	Osimertinib (i.p) + Rabeprazole (p.o.)
AUC_(0–t)_ (μg/L·h)	160.690 ± 28.593	240.983 ± 27.971***
AUC_(0–∞)_ (μg/L·h)	222.818 ± 43.503	279.758 ± 30.903*
MRT_(0–t)_ (h)	10.293 ± 0.735	9.858 ± 0.453
MRT_(0–∞)_ (h)	18.572 ± 3.763	13.477 ± 2.564*
t_1/2z_ (h)	11.070 ± 2.396	7.198 ± 2.976*
T_max_ (h)	5.833 ± 1.169	7.000 ± 2.000
V_z/F_ (L/kg)	145.372 ± 28.071	73.554 ± 31.172**
CL_z/F_ (L/h/kg)	9.255 ± 1.728	7.229 ± 0.870*
C_max_ (μg/L)	13.543 ± 2.965	22.044 ± 4.633**

Note. AUC, area under the blood concentration–time curve; MRT, mean retention time; *t*_1/2z_, elimination half time; *T*_max_, peak time; *V*_z/F_, apparent volume of distribution; *CL*_z/F_, blood clearance; *C*_max_, maximum blood concentration.

*p < 0.05; **p < 0.01; ***p < 0.001 in comparison with the control group.

**TABLE 5:** The main pharmacokinetic parameters of AZ5104 in two groups of rats (*N* = 6).

Parameters	Osimertinib (i.p)	Osimertinib (i.p) + rabeprazole (p.o.)
AUC_(0–t)_ (μg/L·h)	258.478 ± 35.516	375.839 ± 118.065*
AUC_(0–∞)_ (μg/L·h)	311.775 ± 34.419	471.414 ± 164.251
MRT_(0–t)_ (h)	10.253 ± 0.653	10.289 ± 0.763
MRT_(0–∞)_ (h)	14.863 ± 2.136	15.364 ± 2.210
t_1/2z_ (h)	8.379 ± 2.608	8.674 ± 1.685
T_max_ (h)	7.000 ± 2.757	5.667 ± 2.658
V_z/F_ (L/kg)	79.233 ± 30.668	56.896 ± 14.144
CL_z/F_ (L/h/kg)	6.484 ± 0.749	4.822 ± 2.088
C_max_ (μg/L)	19.267 ± 2.541	28.097 ± 10.030

Note. AUC, area under the blood concentration–time curve; MRT, mean retention time; *t*_1/2z_, elimination half time; *T*_max_, peak time; *V*_z/F_, apparent volume of distribution; *CL*_z/F_, blood clearance; *C*_max_, maximum blood concentration.

*p < 0.05, in comparison with the control group.

## Discussion

Lung cancer is one of the most common malignant tumors in the world. Non-small cell lung carcinoma accounts for about 80% of all lung cancers, and the survival rate of most patients is not high (Cao et al., 2021; Sung et al., 2021). Osimertinib is a third-generation highly selective EGFR mutant inhibitor, which has a significant therapeutic effect in patients with non-small cell lung carcinoma (Jänne et al., 2015; Zhao et al., 2018; Rajappa et al., 2019). However, the effects of osimertinib show obvious interindividual differences caused by metabolic enzyme genetic polymorphism. Osimertinib is mainly metabolized by CYP3A4 in the liver and is converted to AZ5104, which also has a certain pharmacological activity (Yates et al., 2016; Zheng et al., 2018). The significant interindividual variations in the activity of CYP3A4 mainly stem from genetic polymorphism, suggesting that CYP3A4 genetic polymorphism has a significant influence on the metabolism of osimertinib (Zanger et al., 2008; Zhou et al., 2011). In view of the widespread use of osimertinib, a wide range of adverse reactions, and lack of reports about the effects of CYP3A4 variants on osimertinib, we used wild-type CYP3A4.1 as a control to assess the catalytic activities of other 26 CYP3A4 variants in the metabolism of osimertinib *in vitro*.

In the incubation system, many variants (CYP3A4.2, 5, 7, 8, 10, 11, 12, 13, 14, 16, 17, 18, and 20) showed lower catalytic activities relative to CYP3A4.1. As previous research has reported, CYP3A4.20 cannot be incorporated into heme and is recognized as nonfunctional; thus, it has no catalytic activities for osimertinib. Similarly, two variants that were not included in our study (CYP3A4.6 and CYP3A4.30) prematurely terminate codons and also have no catalytic activity (Westlind-Johnsson et al., 2006; Apellániz-Ruiz et al., 2015; Hu et al., 2017; Lin et al., 2019). CYP3A4.17 also showed weak activity, which is similar to the results of brexpiprazole and regorafenib (Li et al., 2019; Chen et al., 2020). Thus, patients with these variants should be classified as poor metabolizers, and more attention should be paid to avoid serious adverse reactions.

Seven variants (CYP3A4.3, 15, 28, 29, 32, 33, and 34) showed higher catalytic activities than CYP3A4.1. Except for CYP3A4.3, the *V*_*max*_ of the other variants increased significantly, which may be the main reason for the increase in catalytic activities. For CYP3A4.3, the decrease in *K*_*m*_ is the main reason for the increase in catalytic activities. Patients with these variants should be classified as strong metabolizers, and more attention should be paid to avoid subtreatment. Although AZ5104 also has pharmacological activity, its concentration is only 10% of the substrate (Yates et al., 2016; Zheng et al., 2018). The remaining CYP3A4 variants (CYP3A4.4, 9, 19, 23, 24, and 31) showed similar catalytic activities to CYP3A4.1, as reflected in the lack of significant differences in their *V*_*max*_ and *K*_*m*_. Taken together, our data suggest that CYP3A4 gene polymorphism does have varying degrees of influence on the metabolism of osimertinib. Although the experiment was only verified *in vitro*, it can still provide a reference for conducting clinical experiments.

Due to various complications, cancer patients often use combinations of multiple drugs, such as antidepressants, antiviral drugs, antibacterial drugs, and gastrointestinal drugs, which can easily lead to DDIs (Weiss et al., 2019). In this study, we examined the effects of 114 screened drugs on the metabolism of osimertinib *in vitro* and found that 16 drugs had a strong inhibitory effect on osimertinib (inhibition degree over 80%). Among these, we found that the PPIs (omeprazole, lansoprazole, and rabeprazole) showed a strong inhibitory effect on osimertinib. PPIs can effectively inhibit the secretion of gastric acid by inhibiting the activity of H^+^/K^+^-ATPase and are commonly used in the treatment of gastrointestinal diseases (Abuhelwa et al., 2019; Ochoa et al., 2020). However, PPIs are also the substrates of P450 and are usually metabolized by CYP2C19 and CYP3A4 (Ishizaki and Horai, 1999; Kim et al., 2003; Jana et al., 2018). PPIs and osimertinib share the same metabolic pathways, and previous studies have shown that PPIs have different degrees of inhibitory effects on CYP2C19 and CYP3A4, which increases the possibility of DDIs (Li et al., 2004; Liu et al., 2005). In view of the potential of osimertinib in the field of anticancer drugs and the extensive use of PPIs in clinical settings, it is meaningful to study the interaction between the two kinds of drugs.

*In vitro*, we mainly analyzed the effects of rabeprazole on the metabolism of osimertinib. The results showed that rabeprazole had a strong inhibitory effect on osimertinib in both RLM and HLM in a mixed way. In addition, we partially studied omeprazole and lansoprazole, and the results were similar to those of rabeprazole. Interestingly, as a third-generation PPI, rabeprazole, is rarely metabolized by P450 enzymes in the liver, so it is considered that its possibility of interaction is rather low. However, we still found that it had a strong inhibitory effect on osimertinib, which is primarily metabolized by CYP3A4. The reason is probably that a small part of rabeprazole is still metabolized by CYP3A4, which has a certain competitive binding effect with osimertinib. Moreover, previous reports have shown that rabeprazole thioether, a major metabolite of rabeprazole, has a stronger inhibitory potency to the activity of CYP3A4 than rabeprazole (Ishizaki and Horai, 1999; Li et al., 2004; Shirasaka et al., 2013). This may provide some explanations for the inhibition of osimertinib by rabeprazole.

To further study the interaction between PPIs and osimertinib, we carried out related experiments in SD rats. We found that when osimertinib and PPIs were both administered orally, the AUC_(0–t)_, AUC_(0–∞)_, and C_max_ of osimertinib decreased to varying degrees, with no significant differences in AZ5104. These findings show that rabeprazole actually reduces the exposure to osimertinib in rats. When osimertinib was administered by intraperitoneal injection and rabeprazole was administered orally, the AUC_(0–t)_, AUC_(0–∞)_, and C_max_ of osimertinib and AZ5104 increased to varying degrees, indicating that rabeprazole can inhibit the metabolism of osimertinib *in vivo*.

We believe that the underlying mechanism involves the inhibition of gastric acid secretion and increase in pH by PPIs, which influences the absorption and blood exposure to osimertinib. This is consistent with some related reports (Vishwanathan et al., 2018; Yasumuro et al., 2018; Lima et al., 2021). When osimertinib is administered by intraperitoneal injection, most of it is directly absorbed into the blood, so the effect of PPIs to inhibit gastric acid secretion may have little influence on osimertinib. Therefore, to avoid drug interactions, the two kinds of drugs should not be administered orally at the same time. In addition, we found that the concentration of osimertinib after oral administration was much higher than that after intraperitoneal injection, which can provide a reference for the formulation of this kind of drug.

In conclusion, this study evaluated the effects of CYP3A4 gene polymorphism and drug interactions, especially the PPIs, on the metabolism of osimertinib. According to our results, CYP3A4 gene polymorphism does have different effects on the metabolism of osimertinib, which can provide some reference for the subsequent establishment of the genotype–phenotype relationship in a clinical setting. In addition, we found that rabeprazole had a strong inhibitory effect on osimertinib in RLM and HLM, with a competitive and non-competitive mixed mechanism. *In vivo*, due to the acid-inhibiting effect of PPIs, their influence on the absorption, rather than on the metabolism, of osimertinib may be stronger, leading to the low concentration of osimertinib in the blood. Moreover, the concentration of osimertinib can reach a higher level by oral administration than by intraperitoneal injection. As osimertinib and PPIs are widely used in clinical practice, our research can provide a precise application basis for the combined use of osimertinib and PPIs.

## Data Availability

The raw data supporting the conclusions of this article will be made available by the authors, without undue reservation.

## Abbreviations

AUC, area under the blood concentration–time curve; CLz/F, blood clearance; C_max_, maximum blood concentration; CYP450, cytochrome P450; DDIs, drug–drug interactions; DAS, drug and statistics; EGFR, epidermal growth factor receptor; HLM, human liver microsomes; IC_50_, half-maximal inhibitory concentration; K_i_, inhibition constant; K_m_, Michaelis–Menten constant; MRT, mean retention time; NADPH, nicotinamide adenine dinucleotide phosphate; PPIs, proton pump inhibitors; RLM, rat liver microsomes; SPSS, Statistical Package for Social Sciences; T_max_, peak time; t_1/2z_, elimination half time; UPLC-MS/MS, ultra-performance liquid chromatography–tandem mass spectrometry; V_max_, maximum velocity of the reaction; V_z/F_, apparent volume of distribution.
