# Supplementary material for: The Influence of CYP3A4 Genetic Polymorphism and Proton Pump Inhibitors on Osimertinib Metabolism
Source: Front Pharmacol. 2022 Mar 10;13:794931. doi: 10.3389/fphar.2022.794931 (PMC8960255; doi:10.3389/fphar.2022.794931)
Supplement: Supplementary file 1 [file DataSheet1.docx]

***Supplementary information***

**The influence of CYP3A4 genetic polymorphism and proton pump inhibitors on Osimertinib metabolism**

Supplementary Figures: 4

Supplementary Tables: 6


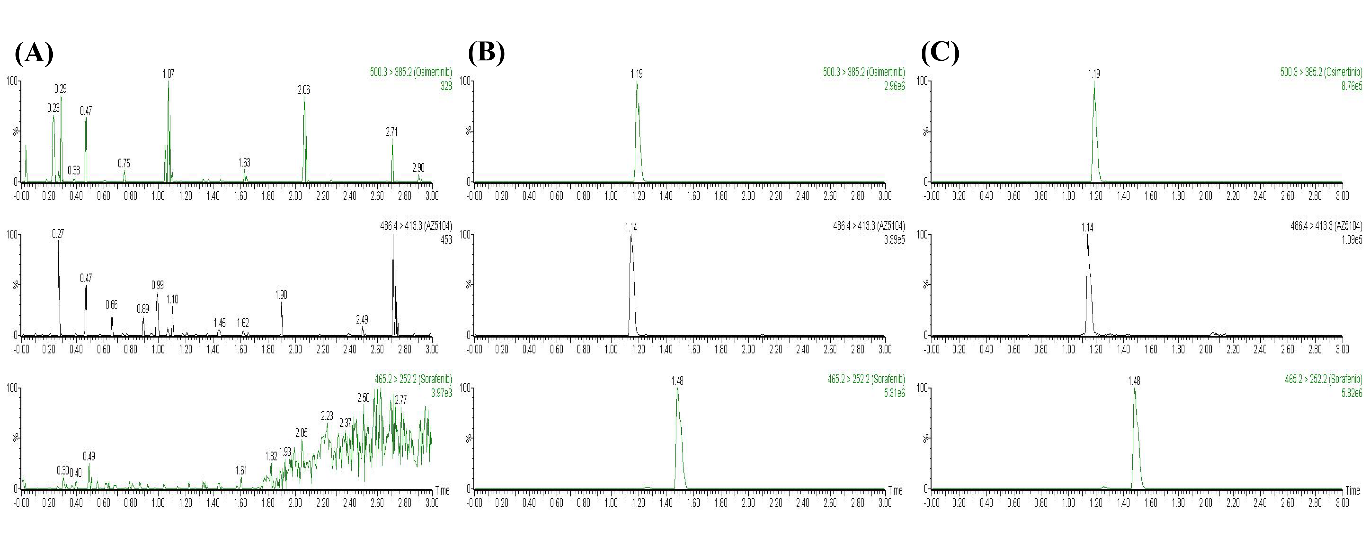


**Supplementary Figure S1.** UPLC-MS/MS chromatographs of osimertinib, AZ5104 and sorafenib (IS).(A) Blank plasma sample. (B) Blank plasma spiked with 200ng/mL osimertinib, 200ng/mL AZ5104 and 100ng/mL sorafenib. (C) Rat plasma sample at 3h after the administration of osimertinib.


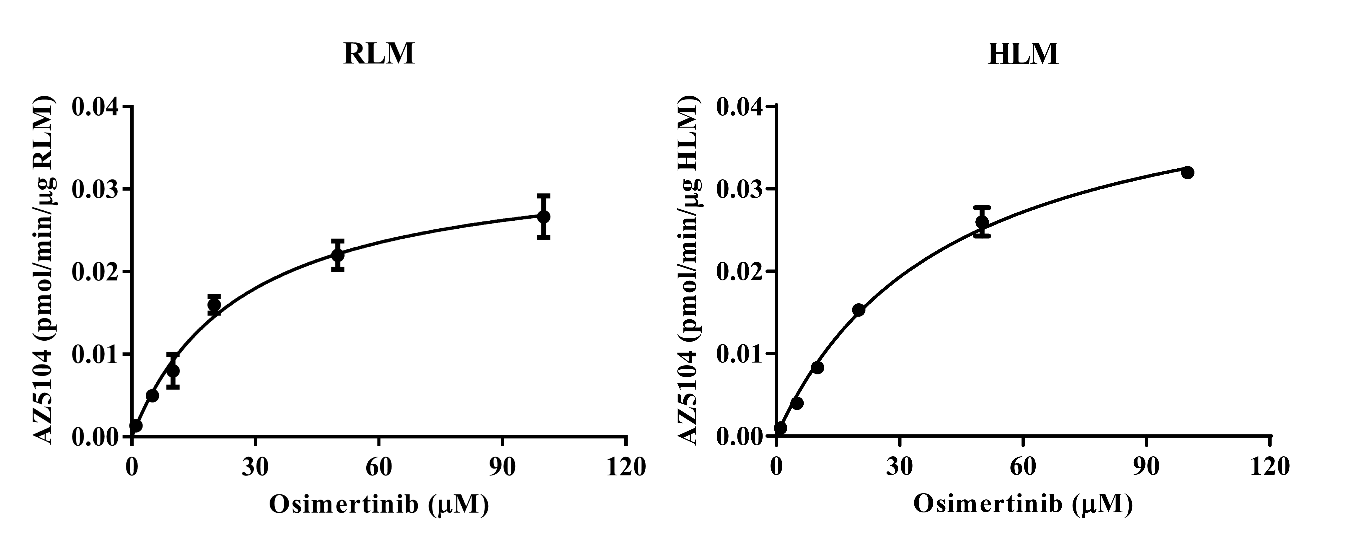


**Supplementary Figure S2.** Michaelis–Menten curves for osimertinib in RLM and HLM, respectively. Data are presented as the means ± SD, n=3.


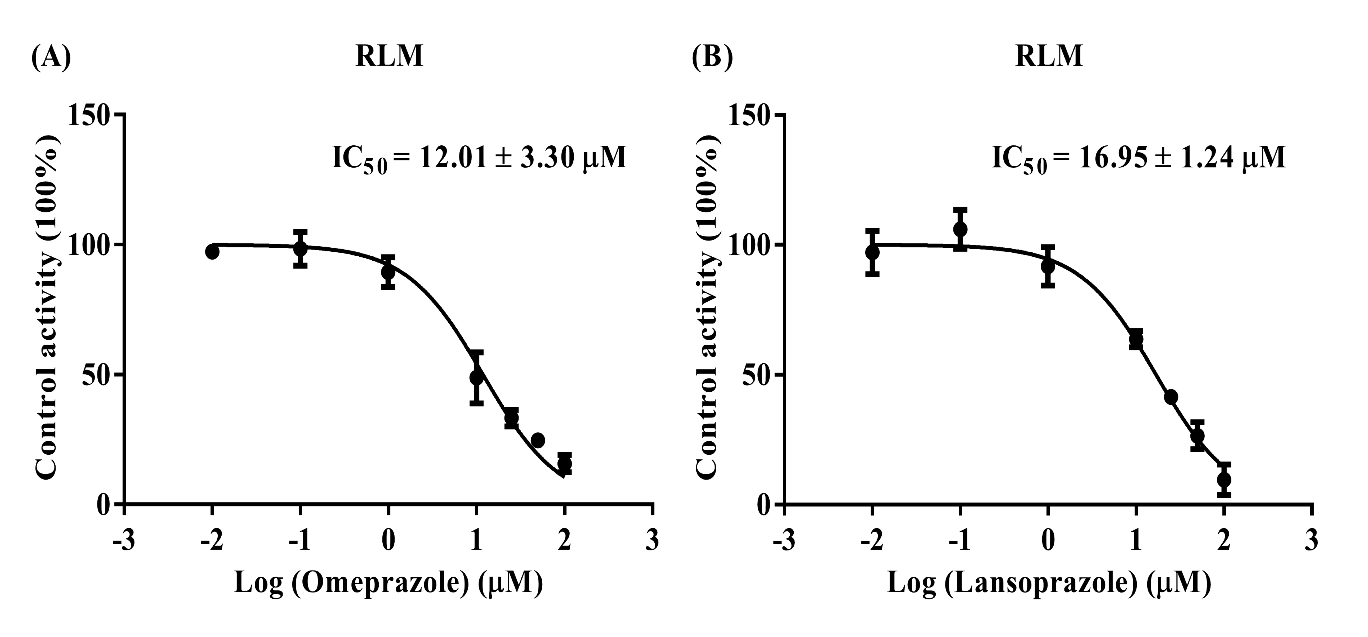


**Supplementary Figure S3.** Various concentrations (0.01, 0.1, 1, 10, 25, 50, 100μM) of (A) omeprazole and (B) lansoprazole for half-maximal inhibitory concentration (IC50) in the activity of RLM, respectively. Data are presented as the means ± SD, n=3.


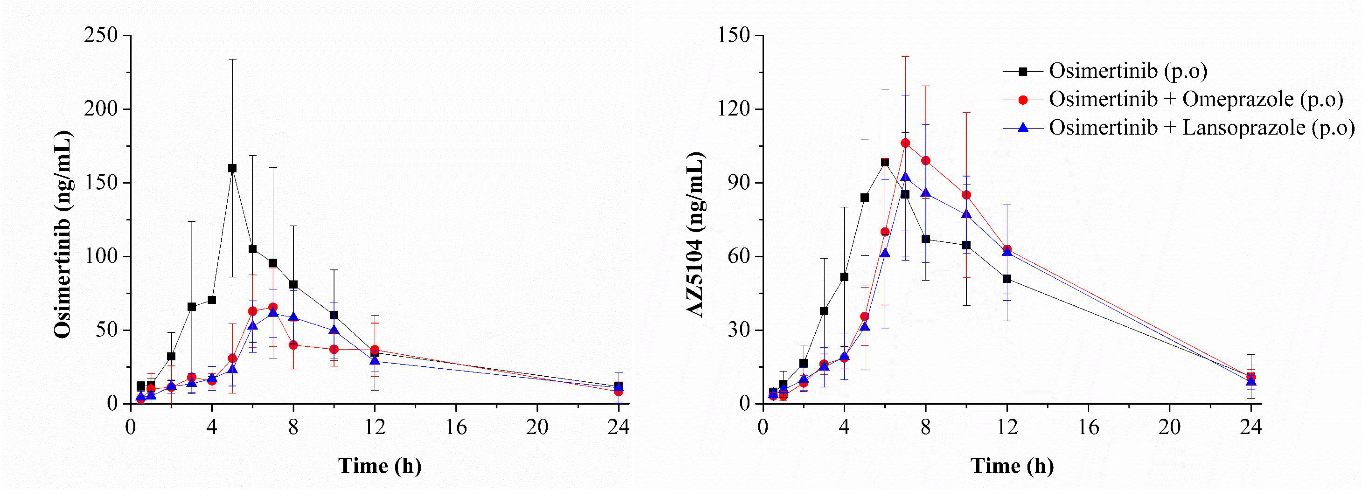


**Supplementary Figure S4.** Mean concentration-time curve of osimertinib and AZ5104 in three groups: osimertinib (p.o), osimertinib (p.o) with omeprazole (p.o) and osimertinib (p.o) with lansoprazole (p.o). Data are presented as the means ± SD, n=6.

**Supplementary Table S1.** The information about the 114 drugs.

| Name | CAS | company |
| --- | --- | --- |
| Toremifene citrate | 89778-27-8 | Shanghai Canspec Scientific Instruments Co., Ltd |
| Diphenhydramine Hydrochloride | 147-24-0 | Shanghai Canspec Scientific Instruments Co., Ltd |
| Terfenadine | 50679-08-8 | Shanghai Canspec Scientific Instruments Co., Ltd |
| Nintedanib | 656247-17-5 | Shanghai Canspec Scientific Instruments Co., Ltd |
| Zopiclone | 43200-80-2 | Shanghai Canspec Scientific Instruments Co., Ltd |
| Phenacetin | 62-44-2 | Shanghai Canspec Scientific Instruments Co., Ltd |
| Modafinil | 68693-11-8 | Shanghai Canspec Scientific Instruments Co., Ltd |
| Trazodone hydrochloride | 25332-39-2 | Shanghai Canspec Scientific Instruments Co., Ltd |
| Sertraline hydrochloride | 79559-97-0 | Shanghai Canspec Scientific Instruments Co., Ltd |
| Mirtazapine | 85650-52-8 | Shanghai Canspec Scientific Instruments Co., Ltd |
| Citalopram hydrobromide | 59729-32-7 | Shanghai Canspec Scientific Instruments Co., Ltd |
| Isavuconazole | 241479-67-4 | Shanghai Canspec Scientific Instruments Co., Ltd |
| Luliconazole | 187164-19-8 | Shanghai Canspec Scientific Instruments Co., Ltd |
| Ketoconazole | 65277-42-1 | Shanghai Canspec Scientific Instruments Co., Ltd |
| Azithromycin | 83905-01-5 | Shanghai Canspec Scientific Instruments Co., Ltd |
| Itraconazole | 84625-61-6 | Shanghai Canspec Scientific Instruments Co., Ltd |
| Fluconazole | 86386-73-4 | Shanghai Canspec Scientific Instruments Co., Ltd |
| Erythromycin | 114-07-8 | Shanghai Canspec Scientific Instruments Co., Ltd |
| Posaconazole | 171228-49-2 | Shanghai Canspec Scientific Instruments Co., Ltd |
| Tricyclazole | 41814-78-2 | Shanghai Canspec Scientific Instruments Co., Ltd |
| Sitafloxacin | 127254-12-0 | Shanghai Canspec Scientific Instruments Co., Ltd |
| Nimodipine | 66085-59-4 | Shanghai Canspec Scientific Instruments Co., Ltd |
| Lovastatin | 75330-75-5 | Shanghai Canspec Scientific Instruments Co., Ltd |
| Atorvastatin | 134523-00-5 | Shanghai Canspec Scientific Instruments Co., Ltd |
| Clarithromycin | 81103-11-9 | Shanghai Canspec Scientific Instruments Co., Ltd |
| Rivaroxaban | 366789-02-8 | Shanghai Canspec Scientific Instruments Co., Ltd |
| Metoprolol tartrate | 56392-17-7 | Shanghai Canspec Scientific Instruments Co., Ltd |
| Bosentan | 147536-97-8 | Shanghai Canspec Scientific Instruments Co., Ltd |
| Darusentan | 171714-84-4 | Shanghai Canspec Scientific Instruments Co., Ltd |
| Irbesartan | 138402-11-6 | Shanghai Canspec Scientific Instruments Co., Ltd |
| Candesartan | 139481-59-7 | Shanghai Canspec Scientific Instruments Co., Ltd |
| Nebivolol | 99200-09-6 | Shanghai Canspec Scientific Instruments Co., Ltd |
| Cilostazol | 73963-72-1 | Shanghai Canspec Scientific Instruments Co., Ltd |
| Simvastatin | 79902-63-9 | Shanghai Canspec Scientific Instruments Co., Ltd |
| Warfarin sodium | 129-06-6 | Shanghai Canspec Scientific Instruments Co., Ltd |
| Enasidenib | 1446502-11-9 | Shanghai Canspec Scientific Instruments Co., Ltd |
| Amlodipine | 88150-42-9 | Shanghai Canspec Scientific Instruments Co., Ltd |
| Losartan | 114798-26-4 | Shanghai Canspec Scientific Instruments Co., Ltd |
| Clonidine hydrochloride | 4205-91-8 | Shanghai Canspec Scientific Instruments Co., Ltd |
| Valsartan | 137862-53-4 | Shanghai Canspec Scientific Instruments Co., Ltd |
| Dronedarone hydrochlorid | 141625-93-6 | Shanghai Canspec Scientific Instruments Co., Ltd |
| Brexpiprazole | 913611-97-9 | Shanghai Canspec Scientific Instruments Co., Ltd |
| Carbamazepine | 298-46-4 | Shanghai Canspec Scientific Instruments Co., Ltd |
| Clonazepam | 1622-61-3 | Shanghai Canspec Scientific Instruments Co., Ltd |
| Ramelteon | 196597-26-9 | Shanghai Canspec Scientific Instruments Co., Ltd |
| Loperamide hydrochloride | 34552-83-5 | Shanghai Canspec Scientific Instruments Co., Ltd |
| Omarigliptin | 1226781-44-7 | Shanghai Canspec Scientific Instruments Co., Ltd |
| Glibenclamide | 10238-21-8 | Shanghai Canspec Scientific Instruments Co., Ltd |
| Glimepiride | 93479-97-1 | Shanghai Canspec Scientific Instruments Co., Ltd |
| Nateglinide | 105816-04-4 | Shanghai Canspec Scientific Instruments Co., Ltd |
| Chlorpropamide | 94-20-2 | Shanghai Canspec Scientific Instruments Co., Ltd |
| Repaglinide | 135062-02-1 | Shanghai Canspec Scientific Instruments Co., Ltd |
| Metformin hydrochloride | 15537-72-1 | Shanghai Canspec Scientific Instruments Co., Ltd |
| Trelagliptin succinate | 1029877-94-8 | Shanghai Canspec Scientific Instruments Co., Ltd |
| Tolbutamide | 64-77-7 | Shanghai Canspec Scientific Instruments Co., Ltd |
| Quinidine | 56-54-2 | Shanghai Canspec Scientific Instruments Co., Ltd |
| Anagrelide | 68475-42-3 | Shanghai Canspec Scientific Instruments Co., Ltd |
| Captopril | 62571-86-2 | Shanghai Canspec Scientific Instruments Co., Ltd |
| Isopropiram | 52373-67-8 | Shanghai Canspec Scientific Instruments Co., Ltd |
| Tapentadol Hydrochloride | 175591-09-0 | Shanghai Canspec Scientific Instruments Co., Ltd |
| Nefopam hydrochloride | 23327-57-3 | Shanghai Canspec Scientific Instruments Co., Ltd |
| Parecoxib | 198470-84-7 | Shanghai Canspec Scientific Instruments Co., Ltd |
| Lopinavir | 192725-17-0 | Shanghai Canspec Scientific Instruments Co., Ltd |
| Ritonavir | 155213-67-5 | Shanghai Canspec Scientific Instruments Co., Ltd |
| Darunavir | 206361-99-1 | Shanghai Canspec Scientific Instruments Co., Ltd |
| Oseltamivir phosphate | 204255-11-8 | Shanghai Canspec Scientific Instruments Co., Ltd |
| Nevirapine | 129618-40-2 | Shanghai Canspec Scientific Instruments Co., Ltd |
| Dexamethasone | 50-02-2 | Shanghai Canspec Scientific Instruments Co., Ltd |
| Valdecoxib | 181695-72-7 | Shanghai Canspec Scientific Instruments Co., Ltd |
| Meloxicam | 71125-38-7 | Shanghai Canspec Scientific Instruments Co., Ltd |
| Flurbiprofen | 5104-49-4 | Shanghai Canspec Scientific Instruments Co., Ltd |
| Celecoxib | 169590-42-5 | Shanghai Canspec Scientific Instruments Co., Ltd |
| Trandolapril | 87679-37-6 | Shanghai Canspec Scientific Instruments Co., Ltd |
| Acetaminophen | 103-90-2 | Shanghai Canspec Scientific Instruments Co., Ltd |
| Valdecoxib | 181695-72-7 | Shanghai Canspec Scientific Instruments Co., Ltd |
| Rabeprazole | 117976-89-3 | Shanghai Canspec Scientific Instruments Co., Ltd |
| Omeprazole | 73590-58-6 | Shanghai Canspec Scientific Instruments Co., Ltd |
| Paliperidone | 144598-75-4 | Shanghai Canspec Scientific Instruments Co., Ltd |
| Tropisetron | 89565-68-4 | Shanghai Canspec Scientific Instruments Co., Ltd |
| Tacrolimus | 104987-11-3 | Shanghai Canspec Scientific Instruments Co., Ltd |
| Rifampicin | 13292-46-1 | Shanghai Canspec Scientific Instruments Co., Ltd |
| Sulfaphenazole | 526-08-9 | Shanghai Canspec Scientific Instruments Co., Ltd |
| Rosuvastatin calcium | 147098-20-2 | Shanghai Canspec Scientific Instruments Co., Ltd |
| Ticagre | 274693-27-5 | Shanghai Canspec Scientific Instruments Co., Ltd |
| Lansoprazole | 103577-45-3 | Shanghai Canspec Scientific Instruments Co., Ltd |
| Irinotecan hydrochloride | 100286-90-6 | Beijing Sunflower and Technology Development Co., Ltd |
| Ambrisentan | 177036-94-1 | Beijing Sunflower and Technology Development Co., Ltd |
| Dextromethorphan hydrobromide | 6700-34-1 | Beijing Sunflower and Technology Development Co., Ltd |
| AgoMelatine | 138112-76-2 | Beijing Sunflower and Technology Development Co., Ltd |
| Dapoxetine hydrochloride | 129938-20-1 | Beijing Sunflower and Technology Development Co., Ltd |
| Fluoxetine hydrochloride | 56296-78-7 | Beijing Sunflower and Technology Development Co., Ltd |
| Amitriptyline hydrochloride | 549-18-8 | Beijing Sunflower and Technology Development Co., Ltd |
| Bupropion hydrochloride | 31677-93-7 | Beijing Sunflower and Technology Development Co., Ltd |
| Venlafaxine hydrochloride | 99300-78-4 | Beijing Sunflower and Technology Development Co., Ltd |
| Vortioxetine | 508233-74-7 | Beijing Sunflower and Technology Development Co., Ltd |
| Voriconazole | 137234-62-9 | Beijing Sunflower and Technology Development Co., Ltd |
| Doxorubicin hydrochloride | 25316-40-9 | Beijing Sunflower and Technology Development Co., Ltd |
| Furazolidone | 67-45-8 | Beijing Sunflower and Technology Development Co., Ltd |
| Ofloxacin | 82419-36-1 | Beijing Sunflower and Technology Development Co., Ltd |
| Cefradine | 38821-53-3 | Beijing Sunflower and Technology Development Co., Ltd |
| ciprofloxacin hydrochloride | 93107-08-5 | Beijing Sunflower and Technology Development Co., Ltd |
| Carvedilol | 72956-09-3 | Beijing Sunflower and Technology Development Co., Ltd |
| Glipizide | 29094-61-9 | Beijing Sunflower and Technology Development Co., Ltd |
| Saxagliptin hydrochloride | 709031-78-7 | Beijing Sunflower and Technology Development Co., Ltd |
| Acyclovir | 59277-89-3 | Beijing Sunflower and Technology Development Co., Ltd |
| Loratadine | 79794-75-5 | Tianjin xiensi Biochemical Technology Co., Ltd |
| Norephedrine hydrochloride | 154-41-6 | Tianjin xiensi Biochemical Technology Co., Ltd |
| Chloramphenicol | 56-75-7 | Tianjin xiensi Biochemical Technology Co., Ltd |
| Clozapine | 5786-21-0 | Tianjin xiensi Biochemical Technology Co., Ltd |
| Penciclovir | 39809-25-1 | Tianjin xiensi Biochemical Technology Co., Ltd |
| Ribavirin | 36791-04-5 | Tianjin xiensi Biochemical Technology Co., Ltd |
| Diclofenac | 15307-79-6 | Tianjin xiensi Biochemical Technology Co., Ltd |
| Telmisartan | 144701-48-4 | Shanghai Aladdin Biochemical Technology Co., Ltd. |
| Disopyramid | 22059-60-5 | Shanghai Aladdin Biochemical Technology Co., Ltd. |

**Supplementary Table S2.** Intra-day and inter-day precision and accuracy of osimertinib and AZ5104 in quality control samples (n=6).

| Analyte | Nominal concentration (ng/mL) | Intra-day | | Inter-day | |
| --- | --- | --- | --- | --- | --- |
|  |  | RSD (%) | RE (%) | RSD (%) | RE (%) |
| osimertinib | 0.2 | 1.78 | 8.13 | 2.74 | 7.30 |
|  | 150 | 2.85 | -6.41 | 7.30 | 1.27 |
|  | 400 | 2.01 | -3.90 | 0.49 | -4.02 |
| AZ5104 | 0.2 | 5.69 | 4.71 | 4.12 | 0.45 |
|  | 150 | 1.88 | 5.39 | 2.38 | 6.84 |
|  | 400 | 2.34 | 9.74 | 0.46 | 9.30 |

**Supplementary Table S3.** Stability of osimertinib and AZ5104 in quality control samples in different environments (n=6).

| Analyte | Nominal concentration (ng/mL) | Room temperature  (24 h) | | 4℃  (24 h ) | | Freeze-thaw  (3 cycles) | |
| --- | --- | --- | --- | --- | --- | --- | --- |
|  |  | RSD (%) | RE (%) | RSD (%) | RE (%) | RSD (%) | RE (%) |
| osimertinib | 0.2 | 11.05 | 3.76 | 9.81 | 3.88 | 6.61 | 5.64 |
|  | 150 | 5.83 | 5.40 | 2.81 | 9.49 | 4.63 | 7.27 |
|  | 400 | 3.98 | -5.38 | 2.90 | 2.59 | 7.08 | -1.25 |
| AZ5104 | 0.2 | 7.59 | 9.90 | 10.29 | 9.36 | 9.95 | -1.52 |
|  | 150 | 7.04 | 10.02 | 8.07 | 8.67 | 7.59 | 8.96 |
|  | 400 | 3.72 | -4.69 | 1.60 | 10.21 | 3.17 | 6.94 |

**Supplementary Table S4.** Matrix effect and Extraction recovery of osimertinib and AZ5104 in quality control samples (n=6).

| Analyte | Nominal  concentration (ng/mL) | Matrix effect | | Extraction recovery | |
| --- | --- | --- | --- | --- | --- |
|  |  | Mean ± SD (%) | RSD (%) | Mean ± SD (%) | RSD (%) |
| osimertinib | 0.2 | 90.24±5.26 | 5.80 | 106.61±7.20 | 6.75 |
|  | 150 | 113.16±3.86 | 3.41 | 102.29±1.31 | 1.28 |
|  | 400 | 107.94±8.21 | 7.60 | 102.57±3.66 | 3.57 |
| AZ5104 | 0.2 | 96.84±6.26 | 6.46 | 94.52±9.17 | 9.70 |
|  | 150 | 105.83±8.24 | 7.78 | 110.09±8.00 | 7.26 |
|  | 400 | 105.78±11.58 | 10.95 | 101.00±3.60 | 3.56 |

**Supplementary Table S5.** The main pharmacokinetic parameters of osimertinib in three groups of rats after oral administration (N=6).

| Parameters | Osimertinib | Osimertinib+Omeprazole | Osimertinib+Lansoprazole |
| --- | --- | --- | --- |
| AUC_(0-t)_ (μg/L·h) | 1,105.401±548.833 | 730.240±312.614 | 553.775±229.764* |
| AUC_(0-∞)_ (μg/L·h) | 1,208.097±590.583 | 823.038±353.515 | 689.543±195.231 |
| MRT_(0-t)_ (h) | 8.268±1.148 | 9.515±1.374 | 9.203±2.251 |
| MRT_(0-∞)_ (h) | 10.085±2.744 | 12.076±3.208 | 12.110±2.499 |
| t_1/2z_ (h) | 5.198±2.771 | 6.222±2.134 | 4.533±2.559 |
| T_max_ (h) | 5.333±1.033 | 6.333±0.516 | 6.833±0.753* |
| V_z/F_ (L/kg) | 30.519±14.042 | 56.427±24.532 | 42.479±21.852 |
| CL_z/F_ (L/h/kg) | 4.513±2.040 | 6.684±3.509 | 6.999±2.060 |
| C_max_ (μg/L) | 163.042±66.742 | 72.099±22.503* | 70.079±16.770* |

^*^P < 0.05, in comparison with the control group.

AUC: area under the blood concentration-time curve; MRT: mean retention time; t_1/2z_: elimination half time; T_max_: peak time; V_z/F_: apparent volume of distribution; CL_z/F_: blood clearance; C_max_: maximum blood concentration.

**Supplementary Table S6.** The main pharmacokinetic parameters of AZ5104 in three groups of rats after oral administration (N=6).

| Parameters | Osimertinib | Osimertinib+Omeprazole | Osimertinib+Lansoprazole |
| --- | --- | --- | --- |
| AUC_(0-t)_ (μg/L·h) | 1,044.596±260.978 | 1,148.755±389.023 | 1,006.229±316.643 |
| AUC_(0-∞)_ (μg/L·h) | 1,118.363±301.951 | 1,844.837±1,513.313 | 1,065.011±333.542 |
| MRT_(0-t)_ (h) | 9.537±0.905 | 10.261±0.388 | 10.369±0.315 |
| MRT_(0-∞)_ (h) | 10.912±1.657 | 21.621±24.912 | 11.482±0.546 |
| t_1/2z_ (h) | 5.010±1.197 | 11.527±16.487 | 4.527±0.406 |
| T_max_ (h) | 6.667±1.633 | 7.333±0.516 | 9.333±1.033** |
| V_z/F_ (L/kg) | 30.203±7.879 | 32.803±15.436 | 29.098±6.041 |
| CL_z/F_ (L/h/kg) | 4.267±1.093 | 3.477±1.741 | 4.499±1.077 |
| C_max_ (μg/L) | 103.368±29.188 | 107.294±34.749 | 91.608±26.777 |

^*^P < 0.05, in comparison with the control group.

AUC: area under the blood concentration-time curve; MRT: mean retention time; t_1/2z_: elimination half time; T_max_: peak time; V_z/F_: apparent volume of distribution; CL_z/F_: blood clearance; C_max_: maximum blood concentration.
